# Medication-related osteonecrosis of the jaw without osteolysis on computed tomography: a retrospective and observational study

**DOI:** 10.1038/s41598-023-39755-6

**Published:** 2023-08-09

**Authors:** Yuki Sakamoto, Shunsuke Sawada, Yuka Kojima

**Affiliations:** 1 Department of Dentistry and Oral Surgery, Kansai Medical University Medical Center, 10-15 Fumizono-Cho, Moriguchi, Osaka 570-8507 Japan; 2 Department of Dentistry and Oral Surgery and Oral Care, Kansai Medical University Hospital, Hirakata, Osaka 573-1191 Japan

**Keywords:** Diseases, Medical research

## Abstract

Medication-related osteonecrosis of the jaw (MRONJ) is a refractory osteonecrosis caused by antiresorptive agents such as bisphosphonate and denosumab (DMB). In MRONJ surgery, computed tomography (CT) is generally used to determine the extent of bone resection. However, in some recent MRONJ cases, no abnormal findings were detected on CT. Therefore, we aimed to clarify the characteristics of MRONJ without osteolysis. This retrospective and observational study included 18 patients diagnosed with MRONJ between October 2020 and October 2022 at Department of Dentistry and Oral Surgery, Kansai Medical University Medical Center. In four of 18 patients with MRONJ, no abnormal findings such as osteolysis, separation of sequestrum, and periosteal reaction were observed on CT images at the first visit. All four patients with non-osteolytic MRONJ had malignant tumors and received high-dose DMB, and in the four patients there were no preceding dental infections such as apical lesions or periodontal disease and the trigger of MRONJ was unknown. Surgery was performed in three of the four patients. The extent of bone resection was determined using magnetic resonance imaging and intraoperative gross findings. In the future, it is necessary to establish a method for diagnosing non osteolytic MRONJ and determining the extent of bone resection.

## Introduction

Medication-related osteonecrosis of the jaw (MRONJ) is a refractory osteonecrosis occurring in patients receiving antiresorptive agent (ARA) therapy such as bisphosphonate (BP) and denosumab (DMB). The incidence rate of MRONJ in patients with osteoporosis treated with low-dose agents to prevent fractures and patients with bone metastases of solid tumors or multiple myeloma treated with high-dose agents for bone-related events is 0.043–0.215% and 0.644–2.731%, respectively^1^. Conservative therapies such as local irrigation, antibacterial mouthwash, and antibacterial drug administration have been recommended to treat stage 2 MRONJ^2^. However, many studies have shown that surgical therapy has better outcomes^3–9^, and the 2022 AAOMS position paper states that both conservative and surgical therapies are acceptable at all stages^10^. Moreover, the cure rate for surgical resection is higher with extensive surgery involving the removal of a portion of the surrounding healthy bone than with conservative surgery involving only necrotic bone removal^3,7–9^. However, there are limited studies on the method used to determine the extent of bone resection. The characteristic imaging finding of MRONJ is osteolysis in all stages. The other findings include osteosclerosis, sequestration, and residual extraction of the fossa^10^. Kojima et al.^11^ reported poor surgical results in patients with a periosteal reaction (PR). Soutome et al. also reported that gap-type and irregular-type PR are infectious lesions that should be included in surgical resection^12,13^. We have previously attempted to resect the bone, including the area of osteolysis on computed tomography (CT) and gap-type and irregular-type PR. We then intraoperatively checked the bone resection margins and removed additional bone if there was a change in bone color or minimal marrow hemorrhage. In MRONJ surgery, the extent of bone resection is determined using preoperative CT and other imaging studies. However, we recently experienced some cases of MRONJ with no osteolysis. In the absence of osteolysis, the surgical procedure is selected based on clinical and magnetic resonance imaging (MRI) findings. MRI may overestimate inflammation or may be unclear as to true osteonecrosis. Therefore, this study aimed to clarify the characteristics of MRONJ without osteolysis (non-osteolytic MRONJ).

## Methods

This retrospective and observational study included 18 patients diagnosed with MRONJ between October 2020 and October 2022 at the Oral and Maxillofacial Surgery Department, Kansai Medical University Medical Center. Exclusion criteria was the patient with no panorama X-ray photo and CT at first visit. The diagnostic criteria for MRONJ are “exposed bone or bone that can be probed through an intraoral or extraoral fistula in the maxillofacial region that has persisted for > 8 weeks” according to the AAOMS 2022 Position Paper^10^. In addition, MRONJ was diagnosed in ARA-treated patients when osteonecrosis was suspected based on clinical and imaging findings, even in the absence of bone exposure or a fistula reaching the bone.

The following variables were investigated: sex, age, site, MRONJ stage, primary disease, type of ARA, administration period, drug holiday after our department visit, diabetes, corticosteroids use, bone exposure, fistula, CT findings (separation of sequestrum, osteolysis, and periosteal reaction), dental infection source, treatment method, and treatment outcome. MRI findings were also investigated in cases in which MRI was performed. Differences between the characteristics of osteolytic MRONJ and non-osteolytic MRONJ were analyzed using Fisher’s exact test or the Mann–Whitney U test. The cure rate was illustrated using the Kaplan–Meier method and analyzed using the log-rank test. The factors related to treatment outcomes were analyzed using Cox regression. All statistical analyses were performed using SPSS ver. 26 (Japan IBM Co., LTD), and two-sided *p* < 0.05 was considered significant.

The study protocol conformed to the ethical guidelines of the Declaration of Helsinki and Ethical Guidelines for Medical and Health Research involving Human Subjects by the Ministry of Health, Labour and Welfare of Japan. Ethical approval was obtained from the institutional review board of Kansai Medical University Medical center (2022048). The research plan was published on the homepage of the hospital according to the instructions of the institutional review board, highlighting the guaranteed opt-out opportunity because this was a retrospective study. The requirement for informed consent was waived by Kansai Medical University Medical center ethical committee.

## Results

### Patient characteristics

Table 1 shows the background factors of the included patients (n = 18). There were four men and 14 women, with an average age of 76.2 ± 7.33 years. The primary diseases were osteoporosis in 11 patients and malignant tumors in seven patients. Eight patients were treated with BP, nine patients were treated with DMB, and one patient was treated with both drugs.

**Table 1:** Patients characteristics.

Variable	Number of patients/mean ± SD
Sex	
Male	4
Female	14
Age (year)	76.2 ± 7.33
Primary disease	
Osteoporosis	11
Malignant disease	7
MRONJ site	
Upper jaw	6
Lower jaw	12
Antiresorptive agent	
BP	8
DMB	10
Duration of administration (month)	25.8 ± 19.3
Drug holiday	
(−)	12
4 months	2
12 months	4
Diabetes	
(−)	11
(+)	7
Corticosteroid use	
(−)	14
(+)	4
Exposed bone	
(+)	11
(−), fistula (+)	6
(−), fistula (−)	1
Trigger	
Apical lesion	2
Periodontal disease	1
Tooth extraction	3
Dentoalveolar surgery	1
Pericoronitis	1
Peri-implantitis	1
Traumatic ulcer	1
Unknown	8
Separation of sequester	
(−)	15
(+)	3
Osteolysis	
(−)	4
(+)	14
Periosteal reaction	
(−)	17
(+)	1
Treatment methods	
Non-surgical therapy	1
Conservative surgery	5
Extensive surgery	12

MRONJ: medication-related osteonecrosis of the jaw, SD: standard deviation, BP: bisphosphonate, DMB: denosumab.

Seventeen of the 18 patients had bone exposure or fistula reaching the bone surface, but one patient had neither bone exposure nor fistula. As a trigger for the onset of MRONJ, 10 patients were considered to have preceding local factors such as apical lesions, periodontal disease, peri-implantitis, and post-extraction. In the remaining eight patients, no teeth could be the source of infection near the site of MRONJ and the onset was unknown. Seven patients had diabetes and four patients were using steroids. CT findings showed sequestrum separation in three patients and periosteal reaction in one patient. Osteolysis was observed in 14 patients, while no osteolysis (non-osteolytic MRONJ) was observed in four patients.

### Non-osteolytic MRONJ

**Figure 1:**
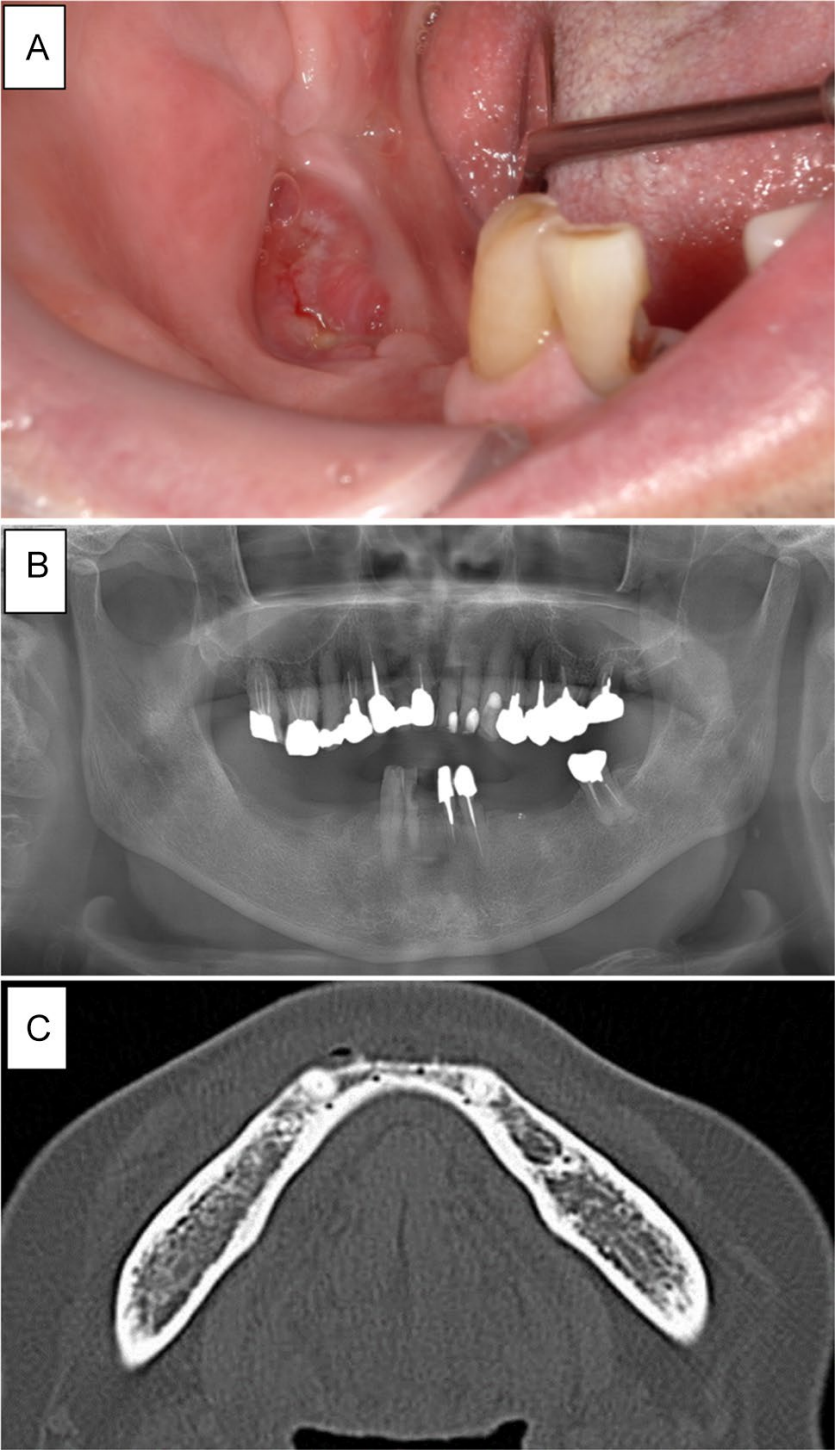
Intraoral and radiographic findings at the initial visit in case 1. (**A**) Exposure of the alveolar bone and redness of the surrounding gingiva are observed. (**B**) Panoramic radiograph showing no abnormal findings except for mild smooth bone resorption on the exposed bone surface. (**C**) Computed tomography showing no abnormal findings in the right mandible’s cortical bone or bone marrow.

**Figure 2:**
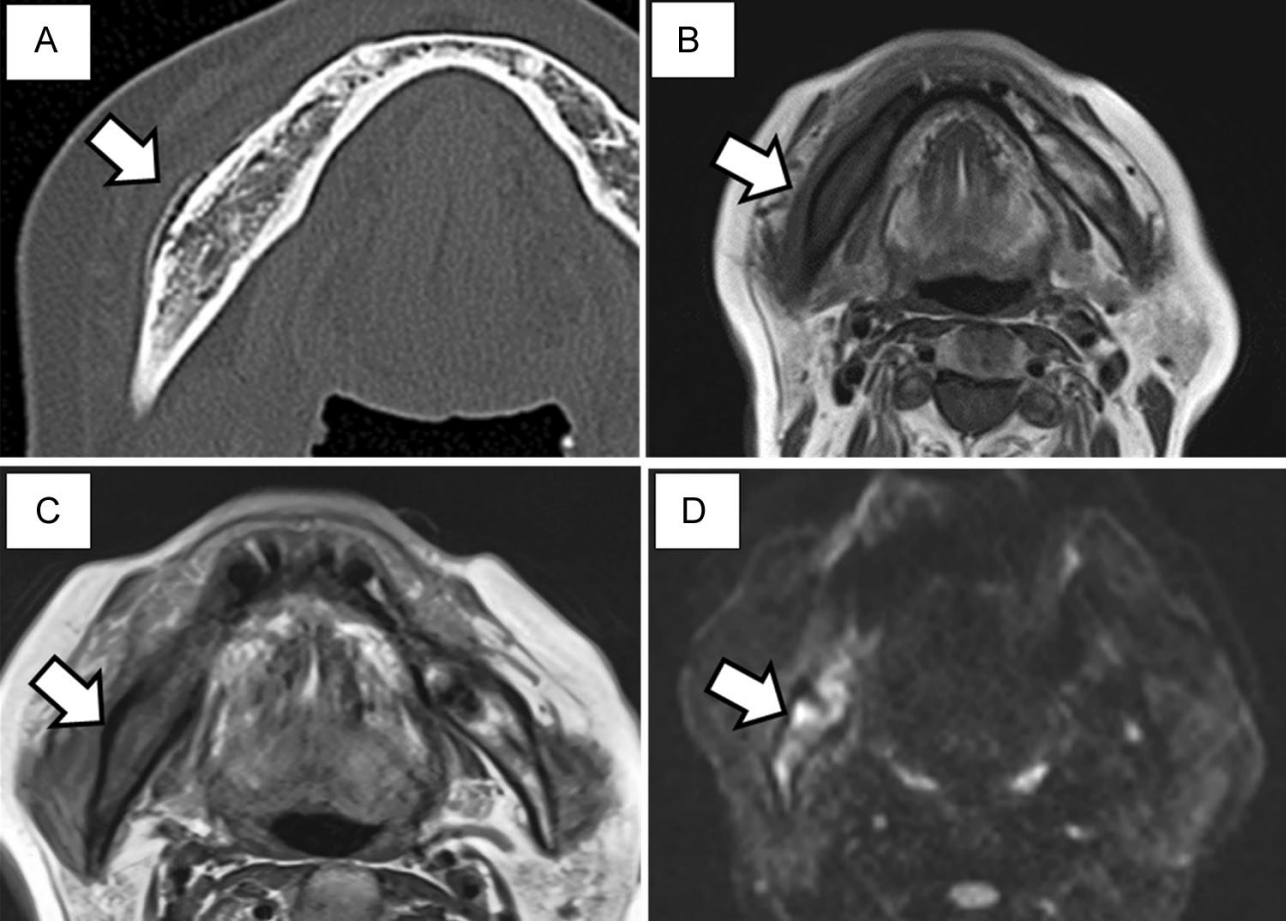
Imaging findings after 4 months. (**A**) An extensive gap-type periosteal reaction is observed on the buccal side of the right mandible. Magnetic resonance imaging. (**B**) Bone marrow signal of the right mandible is reduced in T1-weighted imaging. Low signal continuity is observed in the cortical bone. (**C**) A high signal in the soft tissue around the right mandible and decreased bone marrow signal are observed in T2WI images. (**D**) A high signal is observed from the right mandible to the condyle and in the surrounding soft tissue on T2 short tau inversion recovery imaging.

**Figure 3:**
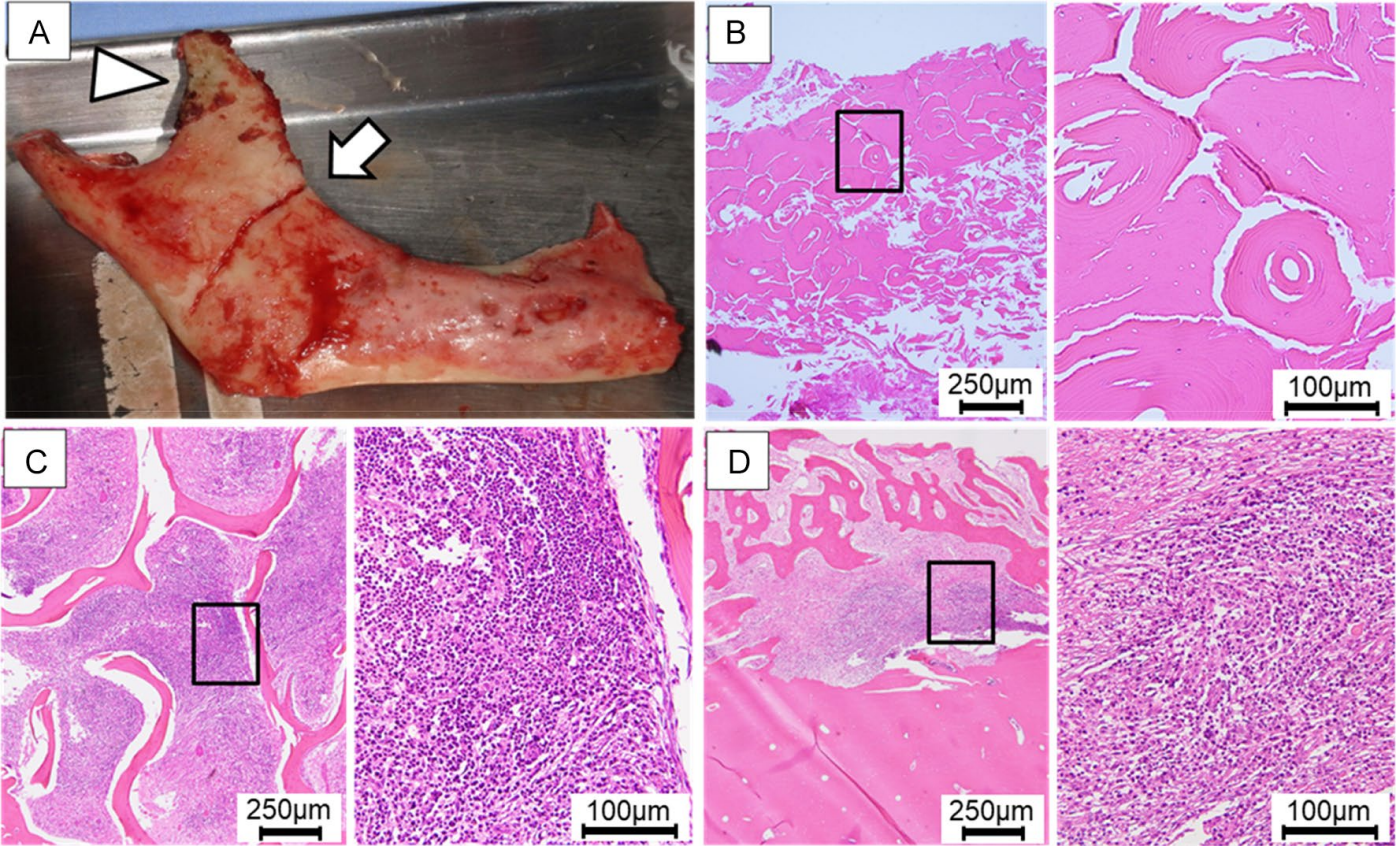
Gross and histological findings of the excised material. (**A**) First, segmental mandibulectomy is attempted (arrow), but a black discoloration is observed in the muscle process (arrowhead); therefore, hemi-mandibulectomy is performed. (**B**) In the cortical bone area, there is osteocyte dropout of lacunae indicating non-vital bone/sequestrum, and the addition of cortical-type bone is observed (HE stain). (**C**) In the bone marrow cavity area, mixed acute and chronic inflammation (with abscess formation) is present (HE stain). (**D**) In the periosteal reaction area, inflammatory granulation tissue is observed between the cortical and new bone (HE stain).

A summary of the four patients with non-osteolytic MRONJ is shown in Table 2. Case 1 involved a 75-year-old man receiving DMB for 9 months for bone metastasis from prostate cancer. The patient was referred to our department with complaints of pain in the mandibular molar area. Intraoral examination revealed bone exposure of the mandibular molar region. However, abnormal finding was only found slight osteosclerosis on panoramic radiography and CT (Fig. 1). The patient was diagnosed with stage 2 MRONJ and received conservative treatment including local irrigation of the exposed bone and use of an oral mouthwash. However, a cutaneous fistula developed in the submandibular region 3 months later. He was treated with oral antibiotics (amoxicillin 250 mg × 3 times a day for one month), but submandibular cellulitis developed. Then, the patient was hospitalized and administered intravenous antibiotics. A bacteriological examination of the abscess detected α-streptococci, which was sensitive to SBT/PIPC. SBT/PIPC 3 g × 2 times a day was used for 1 week was administered. CT showed no bone resorption. However, an extensive gap-type periosteal reaction was observed (Fig. 2). Therefore, we decided to perform extensive surgery because the symptoms worsened despite conservative treatment. However, it was difficult to determine the extent of necrotic bone because no bone resorption was observed on CT. MRI showed extensive changes in the bone marrow signals (Fig. 2); therefore, surgery was initiated with a planned segmental mandibulectomy. Gross findings during surgery included extensive color change on the surface of the mandible and black discoloration of the coronoid process of the mandible. Therefore, segmental mandibulectomy was changed to hemi-mandibulectomy (Fig. 3). Histopathological examination revealed the disappearance of osteocytes in the bone lumen, abscesses, and inflammatory granulation tissue in the bone marrow cavity. Osteoclasts and Howship’s fossa were not observed. At the site of the PR on CT, granulation tissue with inflammatory cell infiltration, mainly neutrophils, was observed between the new and existing bone (Fig. 3). One year after surgery, the patient was doing well with no recurrence.

**Tabel 2:** Summary of the four patients with non-osteolytic MRONJ.

	Case 1	Case 2	Case 3	Case 4
At first visit				
Clinical features				
Bone exposure	+	+	−	−
Fistula reaching the bone surface	−	−	−	+
Infection symptom	+	+	-	+
CT findings				
Osteolysis	−	−	−	−
Periosteal reaction	−	−	−	−
Before surgery (time from first visit to surgery)	4 months	5 months	9 months	5 months^a^
Clinical features				
Bone exposure	+	+	+	−
Fistula reaching the bone surface	+	+	+	−
Infection symptom	+	+	+	+
CT findings				
Osteolysis	−	−	−	−
Periosteal reaction	+	+	−	+
MR findings of bone marrow				
T1WI	Low	Low	Low	Low
T2WI	Low	Low	Mix	Low
T2 STIR	High	High	High	High

MRONJ: medication-related osteonecrosis of the jaw.

^a^Case 4 did not undergo surgery.

Case 2 pertained to a 66-year-old woman who had been receiving DMB for 2 years because of bone metastasis from breast cancer. She presented to our department with the chief complaint of mandibular pain. Bone exposure was observed in the right mandibular molar area of the oral cavity, and the surrounding gingiva was red. However, panoramic radiography and CT showed no abnormal findings. Although she was diagnosed with stage 2 MRONJ, she refused aggressive treatment and chose conservative treatment. Despite weekly local irrigation and administration of oral antibiotics (Amoxicillin 250 mg × 3 times a day for one month), the exposed bone widened, the pain worsened, nerve palsy appeared, and the patient had difficulty opening his mouth. CT images obtained 5 months later showed no osteolysis, similar to the findings noted at the initial visit. However, a gap-type PR was observed from the mandibular body to the mandibular ramus on CT. T1-weighted imaging (T1WI) showed decreased bone marrow signal in the entire right mandible, and short tau inversion recovery (STIR) imaging showed a high signal in the bone marrow, surrounding soft tissues, and around the medial pterygoid muscle. The patient underwent surgery 5 months after her first visit. The bone surface, where the PR was observed, was rough and swollen. Since there was a change in the bone marrow signal up to the condyle on MRI, the resection range was hemi-mandibular. The mandible showed extensive discoloration due to necrosis, and there was not much bleeding when the mandibular canal was cut. Histopathological findings showed abscesses, actinomycete masses, brown pigment, granulation in the bone marrow cavity, and infiltration of inflammatory cells, mainly neutrophils and plasma cells. No osteoclasts were observed. Two samples were taken from the bone marrow of the premolar and mandibular angles during surgery and analyzed using real-time polymerase chain reaction (PCR), and bacterial DNA was detected in both samples. Eleven months after surgery, the complete mucosal coverage was observed without any signs and symptoms of MRONJ.

Case 3 involved a 70-year-old woman who had been receiving DMB for bone metastasis from breast cancer for 17 months. She was referred to our department with the chief complaint of mandibular pain. There was no bone exposure or fistula, and no bone resorption was observed on the panoramic radiography and CT. The symptoms reduced after oral antibiotic administration (Amoxicillin 250 mg three times a day for one week), and the patient was followed up without further treatment. Six months later, bone exposure to the oral cavity was observed in the same area, and pain and pus discharge were also observed; therefore, tooth extraction and shaving of the sharp edge of the alveolar bone were performed. However, the wound was opened, and the discharge did not disappear. MRI showed a decrease in the bone marrow signal to the region around the inferior margin of the mandible and a high signal on STIR, mainly at the site where osteosynthesis was performed. CT was repeated, but neither osteolysis nor PR was observed. Therefore, the decision was made to repeat surgery. Surgery was performed based on the diagnosis of MRONJ. We decided to make the decision regarding the type of surgery intraoperatively because preoperative CT and MRI findings made it difficult to decide whether to perform marginal mandibulectomy or segmental mandibulectomy plus reconstruction with titanium plate. An intraoral incision was made, and although gray changes in the alveolar bone were observed, they did not extend to the mandibular inferior margin. Therefore, marginal mandibulectomy was performed. The lower right mandibular, which had been scraped previously, was necrotic and discolored. Discolored bone was removed and resected to include MRI hyperintense and hypointense osteonecrosis. Histopathological findings of the excisional pathology revealed inflammatory cell infiltration in the necrotic bone and marrow cavity, and no osteoclasts were observed. At 3 months postoperatively, the patient was doing well and had no recurrence.

Case 4 involved an 80-year-old man who was referred by a urologist for consultation with a small gingival fistula in the left lower mandible that reached the bone. No bone resorption was observed on panoramic radiography and CT. Under local anesthesia, the fistula was incised, the sequestrum of the alveolar bone was removed, and the wound was closed. The fistula disappeared, and the patient’s visits were interrupted. Five months later, the patient returned to the hospital with pain and swelling in the left lower jaw. The patient underwent incisional drainage and antibiotic administration. Bacterial examination of abscess showed α-streptococci and anaerobic, and ABPC/PIPC were sensitive, SBT/PIPC 3 g × 2 times a day was used for 1 week CT showed a PR. MRI showed decreased signal intensity on T1WI and high signal intensity on STIR imaging, as in the other three patients. After the acute inflammation had healed, a residual fistula was observed in the oral cavity; however, according to the patient’s request, surgery was not performed. The patient has been treated repeatedly with antimicrobials for swelling and pain that worsen about almost every 3 months. Fifteen months later, bone exposure and a fistula are present, and there is no improvement in symptoms.

At the first visit, bone exposure or fistula reaching the bone was observed in three of the four cases, but no bone exposure or fistula was observed in one case. No osteolysis or PR was observed in any of the four cases on panoramic radiography or CT, except mild osteosclerosis. Although there was some osteosclerosis, no abnormal findings were observed. Therefore, surgical treatment was not initially performed in all four cases, and follow-up was performed only with conservative treatment. However, the symptoms of infection worsened, and CT performed after 5–9 months showed a PR in three cases, although no osteolysis was observed. MRI showed a low signal intensity on T1WI, low to mixed signal intensity on T2-weighted imaging, and high T2 signal intensity in the bone marrow on STIR imaging.

### Differences between osteolytic MRONJ and non-osteolytic MRONJ

We compared the characteristics of patients with non-osteolytic MRONJ and those with osteolytic MRONJ (Table 3). The primary disease of the four patients with non-osteolytic MRONJ was malignant tumors, which were treated with high-dose DMB, and the trigger was unknown (not a dental infection). Univariate analysis revealed that the non-osteolytic type was significantly more common in patients with malignant tumors as the primary disease who were receiving DMB as the antiresorptive agent. However, multivariate analysis could not be performed owing to the small number of cases.

**Table 3:** The characteristics of patients with non-osteolytic and osteolytic MRONJ.

Variable	Osteolytic MRONJ	Non-osteolytic MRONJ	*p*-value
Sex			
Male	2	2	0.197
Female	12	2	
Age	77.2 ± 7.76	72.5 ± 5.50	
Primary disease			
Osteoporosis	11	0	0.011*
Malignant disease	3	4	
MRONJ site			
Upper jaw	6	0	0.245
Lower jaw	8	4	
Antiresorptive agent			
BP	9	0	0.041*
DMB	5	4	
Duration of administration			
< 1 years	5	1	1.000
≥ 1 years	9	3	
Drug holiday			
(−)	10	2	0.568
(+)	4	2	
Diabetes			
(−)	9	2	1.000
(+)	5	2	
Corticosteroid use			
(−)	12	2	0.197
(+)	2	2	
Exposed bone			
(−)	6	1	0.622
(+)	8	3	
Fistula			
(−)	4	1	1.000
(+)	10	3	
Source of infection			
Odontogenic	10	0	0.023*
Tooth extraction	3	0	
Apical lesion	2	0	
Periodontal disease	1	0	
Dentoalveolar surgery	1	0	
Pericoronitis	1	0	
Peri-implantitis	1	0	
Traumatic ulcer	1	0	
Unknown	4	4	
Separation of sequestrum			
(−)	11	4	1.000
(+)	3	0	
Periosteal reaction			
(−)	14	3	0.222
(+)	0	1	
Treatment methods			
Non-surgical therapy	0	1	0.083
Conservative surgery	5	0	
Extensive surgery	9	3	

MRONJ: medication-related osteonecrosis of the jaw, BP: bisphosphonate, DMB: denosumab.

[*] means that there is a significant difference, showing a p-value of <0.05.

### Treatment outcomes

Surgery was performed in 17 of the 18 patients, excluding one patient with a poor general condition. If the sequestrum was separated, only the sequestrum was removed. If the necrotic bone was not completely separated, resection was performed including the surrounding healthy bone. In the case of non-osteolytic MRONJ, the approximate extent of bone resection was determined by preoperative MRI, and the extent was modified according to intraoperative findings (bone coloration or presence of bleeding). The surgical method was sequestrum resection in five patients, marginal mandibulectomy in 10 patients, and hemi-mandibulectomy in two patients. The treatment outcome was a complete cure in 11 patients, and the cumulative cure rate was 61.1%.

**Figure 4:**
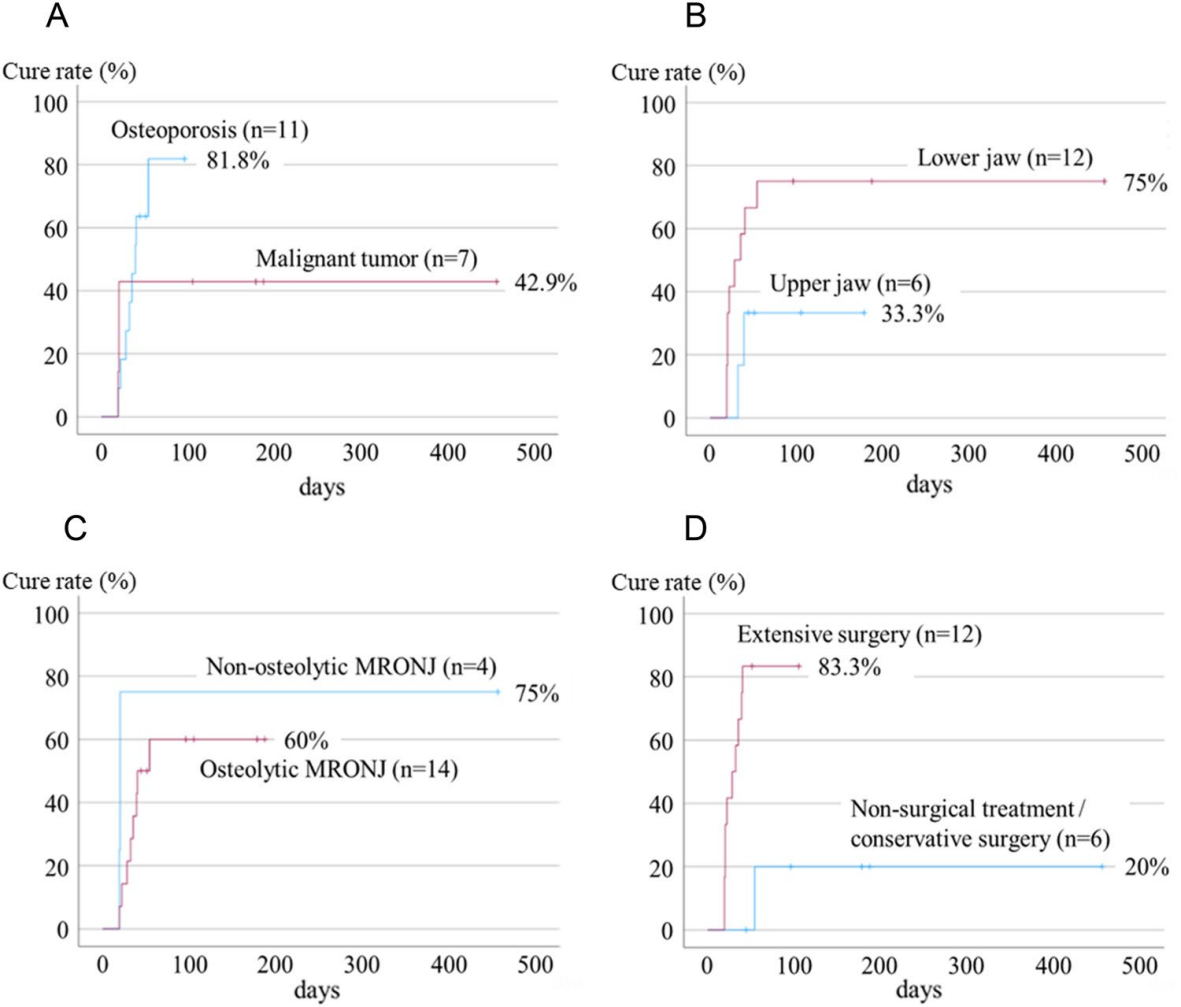
Factors related to treatment outcomes. (**A**) Patients with malignant tumors have a significantly lower cure rate than those with osteoporosis. (**B**) Patients with maxillary lesions have a higher cure rate than those with mandibular lesions, but the difference is insignificant. (**C**) A difference between the cure rate of osteolytic MRONJ and non-osteolytic MRONJ is not found. (**D**) Patients who underwent extensive surgery had a significantly better prognosis than those who underwent non-surgical or conservative surgery. MRONJ, medication-related osteonecrosis of the jaw.

Factors related to treatment outcomes are shown in Table 4 and Fig. 4. Patients with malignant tumors had a lower cure rate than those with osteoporosis (hazard ratio [HR] = 0.565, 95% confidence interval [CI] = 0.146–2.187, *p* = 0.408), and patients with maxillary lesions had a higher cure rate than those with mandibular lesions (HR = 3.210,95% CI = 0.688–14.969, *p* = 0.099). However, the difference was not significant on univariate Cox regression analysis. In addition, the difference in cure rates between osteolytic MRONJ and non-osteolytic MRONJ was unclear (HR = 0.203, 95% CI = 0.107–1.610, *p* = 0.414). Patients who underwent extensive surgery had a significantly better prognosis than those who underwent non-surgical or conservative surgery (HR = 12.234, 95% CI = 1.470–101.840, *p* = 0.021). However, the number of cases was small and multivariate analysis could not be performed; therefore, the details could not be clarified.

**Table 4:** Factors related to the treatment outcomes.

Variable		p-value	HR	95% CI
Sex	Male/female	0.211	0.266	0.033–2.123
Age	(years)	0.725	0.985	0.907–1.070
Primary disease	Malignant disease/osteoporosis	0.408	0.565	0.146–2.187
MRONJ site	Lower jaw/upper jaw	0.099	3.210	0.688–14.969
Antiresorptive agent	DMB/BP	0.636	0.750	0.228–2.465
Duration of administration	(months)	0.171	1.023	0.990–1.056
Duration of drug holiday	(months)	0.544	1.040	0.916–1.180
Diabetes	(+)/(−)	0.230	0.434	0.111–1.695
Corticosteroid use	(+)/(−)	0.246	2.122	0.595–7.564
Exposed bone	(+)/(−)	0.461	1.570	0.472–5.220
Fistula	(+)/(−)	0.431	0.585	0.154–2.218
Source of infection	Unknown/odontogenic	0.414	1.643	0.499–5.412
Separation of sequestrum	(+)/(−)	0.174	2.567	0.659–9.991
Osteolysis	(+)/(−)	0.203	0.414	0.107–1.610
Periosteal reaction	(+)/(−)	0.509	0.043	0.000–496.247
Treatment methods	Extensive surgery/non-surgical or conservative surgery	0.021*	12.234	1.470–101.840

Univariate cox regression analysis.

MRONJ: medication-related osteonecrosis of the jaw; BP: bisphosphonate; DMB: denosumab; HR: hazard ratio, CI: confidence interval.

[*] means that there is a significant difference, showing a p-value of <0.05.

## Discussion

This study shows that DMB-induced MRONJ in patients with cancer is occasionally not accompanied by osteolytic lesions on CT. According to Damm et al.^14^, the skeletal system has a mixture of resting (osteocytes: 85%), resorbing (osteoclasts: 2%), and forming (osteoblasts: 10–12%) surfaces. Osteocytes have a lifespan of several days to 10 years, osteoclasts have a lifespan of 2 weeks, and osteoblasts have a lifespan of 1–3 months. BP and DMB suppress bone resorption through different mechanisms. However, both exert their effects on bone resorption by suppressing osteoclasts. Therefore, impaired bone remodeling is considered a cause of MRONJ development. Approximately 50% of BP taken up in the serum is rapidly excreted by the kidneys within hours, resulting in a rapid drop in its blood levels. However, BP binds specifically to the bone matrix, and BP incorporated into the bone matrix remains in the bone until remodeling, with a half-life of several to 10 years. BP is released from the bone matrix in the acidic environment of resorption pores during bone resorption by osteoclasts and is incorporated into osteoclasts to destroy the osteoclast skeleton and induce apoptosis, thereby inhibiting bone resorption by destroying and inducing apoptosis. Osteoclasts under the influence of BP are large multinucleated osteoclasts detached from the bone surface^15^.

In contrast, DMB is incorporated into the serum circulates in the blood, including the bone marrow vessels. Its half-life in the blood is 25.4 days; unlike BP preparations, it does not exhibit specific binding to the bone matrix or accumulate. Therefore, the action of DMB is considered more reversible than that of BP^16^. Hence, this inhibits the differentiation of osteoclasts into mature osteoclasts and activates them. It also inhibits osteoclast differentiation and survival. Consequently, the total number of osteoclasts under the influence of DMB is decreased, and small immature osteoclasts are observed^17^.

Limones et al. analyzed the incidence, risk ratio, and prognoses of two types of MRONJ (DMB-related and BP-related osteonecrosis of the jaw) in patients with cancer in a systematic review and concluded that DMB was associated with a significantly higher risk of developing MRONJ than BP^18^. These results show that DMB has a stronger inhibitory effect on bone metabolism than BP. We quantified the bone metabolism-related genes ALP, OCN, DMP-1, TRAP, and CTSK in surgically resected MRONJ specimens using real-time reverse transcriptase PCR. We found that bone metabolism was significantly reduced in DMB-induced osteonecrosis of the jaw compared to BP-induced osteonecrosis of the jaw (unpublished data). As described above, BP is taken up by osteoclasts during bone resorption and inhibits osteoclast function. Therefore, a certain degree of bone resorption occurs in patients treated with BP. However, since DMB stops osteoclast differentiation, osteoclasts are not formed and bone resorption is expected to stop completely in DMB-administered cases. Although our report is inconclusive due to the small number of cases, all patients with non-osteolytic MRONJ received high-dose DMB. The reason why non-osteolytic MRONJ is seen in patients associated with DMB may be related to the fact that the inhibitory effect of DMB on bone resorption is stronger than that of BP^19^. Pichardo et al.^20^ reported less cortical bone resorption in DMB than BP, and Favia et al.^21^ reported more advanced stage findings in MRONJ associated with DMB, which may suggest less bone resorption in MRONJ associated with DMB as in our report here, further investigations are required to determine whether non-osteolytic MRONJ occurs only in patients receiving high doses of DMB.

MRONJ is defined as bone exposure or a fistula reaching the bone that has persisted for more than 8 weeks in patients with current or previous ARA therapy^10^. Two of the 4 patients had bone exposure and one had a fistula reaching the bone at the first visit. One patient with neither bone exposure nor fistula subsequently developed bone exposure and fistula. Thus, all four of these cases were diagnosed with MRONJ.

There have been several reports on the diagnosis of osteomyelitis not associated with ARA and MRONJ. Shin et al. reported in a retrospective study of 270 patients, that sequestrum and periosteal reaction were more commonly observed in MRONJ patients than in osteomyelitis, but other CT imaging features, such as trabecular defects, cortical defects, sclerosis, and soft tissue changes showed no significant differences between the two, so they concluded that MRONJ and osteomyelitis are hard to differentiate with images^22^. Wongratwanich et al. suggested that MRI can help detect early bone marrow changes and soft tissue swelling^23^. Stage 0 of MRONJ showed hypointensity on T1WI and hyperintensity on T2WI and STIR. Necrotic bone is expected to show low intensity on all T1WI, T2WI, and STIR. In addition, functional imaging is also useful that the values of bone scan index and standardized uptake value may be predictive of MRONJ progression factors^24^. However, these findings on MRI are also seen in patients with osteomyelitis not related to ARA. De Antoni et al. performed histopathologic searches for MRONJ, osteoradionecrosis (ORN), and osteomyelitis of the jaw (OMJ), and concluded that histopathological aspects of MRONJ, ORN, and OMJ do not permit a conclusive diagnosis^25^. As described above, it is not easy to differentiate MRONJ from osteomyelitis by imaging or histopathology.

Clinically, MRONJ and osteomyelitis are quite different in character. Osteomyelitis is often a reversible lesion with antimicrobial therapy. In contrast, MRONJ is rarely curable with conservative treatment alone^11^ and often requires surgical removal of necrotic bone^3,7,8^. In three of the four cases of non-osteolytic MRONJ in this study, we considered surgery necessary because the symptoms worsened despite antimicrobial therapy for 4 to 9 months after the initial diagnosis. We believe that if an early diagnosis method for non-osteolytic MRONJ is established in the future, minimally invasive surgery will be possible before major resection is required.

In MRONJ surgery, we determined the extent of bone resection mainly based on preoperative CT images. However, there has been an increasing number of cases in which the extent of bone resection has been difficult to determine because of no osteolysis observed on CT. The recent increase in such cases may be due to the fact that patients without osteolysis were not previously diagnosed with MRONJ and were not candidates for surgery. In this study, two non-osteolytic MRONJ cases were advanced cases, and performing hemi-mandibulectomy might have led to favorable treatment outcomes. It is difficult to determine the surgical method for non-osteolytic MRONJ, which may affect the treatment outcomes. In the future, it will be necessary to conduct a multicenter study with a larger number of patients.

This study has some limitations. First, it was a retrospective study with a small number of patients, and the follow-up period was short. Therefore, the lack of sufficient statistical examination makes it unclear whether the results obtained can be generalized. However, this is the first study to show that MRONJ may develop without osteolysis on CT in patients receiving high-dose DMB. In the future, we would like to increase the number of cases and examine the characteristics and treatment methods of non-osteolytic MRONJ.

## Data Availability

The datasets generated and/or analyzed during the current study are available from the corresponding author on reasonable request.
